# HEALTH CARE PROVIDER PERSPECTIVES ON MENTAL HEALTH CARE WITHIN CHRONIC CARE MANAGEMENT: A RURAL STUDY

**DOI:** 10.1093/geroni/igae098.3014

**Published:** 2024-12-31

**Authors:** Elizabeth Punke, Lucas Wall, Barbara Dabrowski, Abby Teply, Katy Richardson, Catherine Carrico, Christine McKibbin

**Affiliations:** University of Wyoming, Laramie, Wyoming, United States; UWYO, Laramie, Wyoming, United States; University of Wyoming, Laramie, Wyoming, United States; University of Wyoming, Laramie, Wyoming, United States; UWYO, Laramie, Wyoming, United States; University of Wyoming, Laramie, Wyoming, United States; University of Wyoming, Laramie, Wyoming, United States

## Abstract

Approximately 59% of older adult Medicare beneficiaries experience two or more chronic conditions. This rate is even higher among those dually-enrolled in Medicare and Medicaid (i.e., 77%; Boersma et al. 2021). In 2015, the Centers for Medicare and Medicaid Services introduced Chronic Care Management (CCM), a value-based care program, to provide reimbursement to practices for care coordination services that support the management of patients with multimorbidity. Importantly, physical and mental health conditions are often interrelated; however, providers often struggle to adequately treat mental health needs in the context of physical illness (Lattie et al., 2020). The purpose of this study was to examine healthcare providers’ perspectives on serving individuals with mental health diagnoses enrolled in CCM within primary care practices. Investigators developed an interview for this qualitative study. Employees of six practices that have implemented CCM participated. Participants completed sociodemographic, professional, and practice history measures, and a qualitative interview. Participants (n = 13) were predominantly White, cisgender females (n = 12; 92%) and represented care coordinators, primary care providers, allied health professionals, and administrators. All participants completed a 60-minute interview, which was audio recorded, transcribed verbatim, and subjected to thematic analysis. Four themes were identified: Types of Chronic Health Conditions Treated, Access to Mental Health Services, Provider Experience and Comfort, and Educational Needs. Participants expressed a willingness to complete mental health training but faced challenges identifying particular training needs. Future training efforts should address foundational knowledge in mental health disorders and strategies to serve people with common mental health comorbidities.

